# Sodium Nitroprusside Improves Bamboo Resistance under Mn and Cr Toxicity with Stimulation of Antioxidants Activity, Relative Water Content, and Metal Translocation and Accumulation

**DOI:** 10.3390/ijms24031942

**Published:** 2023-01-18

**Authors:** Abolghassem Emamverdian, Yulong Ding, James Barker, Guohua Liu, Yang Li, Farzad Mokhberdoran

**Affiliations:** 1 Co-Innovation Center for Sustainable Forestry in Southern China, Nanjing Forestry University, Nanjing 210037, China; 2 Bamboo Research Institute, Nanjing Forestry University, Nanjing 210037, China; 3 School of Life Sciences, Pharmacy and Chemistry, Kingston University, Kingston-upon-Thames KT1 2EE, UK; 4 Department of Mathematical Sciences, Florida Atlantic University, Boca Raton, FL 33431, USA

**Keywords:** bamboo species, nitric oxide, heavy metal stress, plant stress tolerance, plant phytoremediation

## Abstract

Sodium nitroprusside (SNP), as a single minuscule signaling molecule, has been employed to alleviate plant stress in recent years. This approach has a beneficial effect on the biological and physiological processes of plants. As a result, an in vitro tissue culture experiment was carried out to investigate the effect of high and low levels of SNP on the amelioration of manganese (Mn) and chromium (Cr) toxicity in a one-year-old bamboo plant, namely *Pleioblastus pygmaea* L. Five different concentrations of SNP were utilized as a nitric oxide (NO) donor (0, 50, 80, 150, 250, and 400 µM) in four replications of 150 µM Mn and 150 µM Cr. The results revealed that while 150 µM Mn and 150 µM Cr induced an over-generation of reactive oxygen species (ROS) compounds, enhancing plant membrane injury, electrolyte leakage (EL), and oxidation in bamboo species, the varying levels of SNP significantly increased antioxidant and non-antioxidant activities, proline (Pro), glutathione (GSH), and glycine betaine (GB) content, photosynthesis, and plant growth parameters, while also reducing heavy metal accumulation and translocation in the shoot and stem. This resulted in an increase in the plant’s tolerance to Mn and Cr toxicity. Hence, it is inferred that NO-induced mechanisms boosted plant resistance to toxicity by increasing antioxidant capacity, inhibiting heavy metal accumulation in the aerial part of the plant, restricting heavy metal translocation from root to leaves, and enhancing the relative water content of leaves.

## 1. Introduction

In recent years, due to rising anthropogenic activity, both natural resources and the human environment have been contaminated by heavy metals, thus posing a hazard to human society’s health [1]. When plants uptake nutrients from the soil, some heavy metals and non-heavy metals are absorbed through their roots, which can induce fundamental alterations in cellular metabolism [2]. Manganese (Mn) is one of the essential micronutrients with a widespread abundance in China’s forestland and agricultural soils [3]. Although Mn can be used as a plant nutrient and a trace element to promote plant growth and development, excess levels of Mn induce toxicity in plants [4]. Extreme levels of Mn^2+^ can induce a destructive impact on the plant photosynthesis process with generation of ROS through the Fenton reaction, which inhibits the co-factoring role of the element in vital enzymatic reactions and disturbs the photosynthetic processes in plants [5]. Moreover, the preponderance of Mn^2+^ reduces uptake and translocation of some plant essential elements such as potassium (K), calcium (Ca), magnesium (Mg), and iron (Fe) [3], which can directly impact on plant growth and development. Chromium (Cr) can be released into the environment due to its wide use in industries, such as leather processing, mining, wood preservation, petroleum refining, and textile electropainting and manufacturing, etc., and this has resulted in an environmental dilemma [6,7]. Cr and Cr compounds can affect humans through skin contact, eating, breathing, as well as drinking [8]. In China, Cr (VI) ions are the most abundant toxic metal ion resulting from pollution into river waters [9]. In addition, China is the country with the largest production of Cr slag, with 329,000 tons produced and 450,000 tons discharged annually. It is possible that there is stored more than 400 million tons of untreated Cr slags from previous years’ production (State Ministry of Environmental Protection (MEP), 2007) [10]. The chromium in agricultural soil remarkably impacts on the grain quality of plants as well as crop yield, which is a threat to the human food chain [8]. In plants, Cr toxicity reduces plant growth with an impact on chloroplasts and cell membranes and by inducing root cell damage. Chromium, with accompanying chlorosis, influences plant morphology. In a physiological aspect, Chromium can affect mineral nutrition and water retention, enzymatic activities, and pigment content, and could disrupt nitrogen assimilation, transpiration as well as plant photosynthesis and growth [11,12,13]. Chromium leads to the over generation of ROS, which can disrupt the plant redox balance [13]. When a plant is exposed to abiotic stress, it responds typically by generating ROS [14]. Thus, the synthesis of ROS has altered the cellular redox homeostasis, which is the main factor of oxidative stress in plants under heavy metal stress [15]. Additionally, oxidative stress has a debilitating effect on cell damage and plant necrosis, leading to plant cell death [16]. Nitric oxide acts as a signaling molecule in this process by regulating ROS compounds [17].

Nitric oxide is a small signaling molecule and a crucial regulator of the plant life cycle [11], which acts positively during various stages of the plant life cycle, such as germination, flowering, development, and senescence, as well as in the amelioration of abiotic stress [2,18]. Thus, this neutral and tiny redox molecule has a great capacity to spread through the cell membrane and is frequently referred to as a dynamic molecule [11]. SNP, a nitric oxide donor, has been implicated in signaling responses to abiotic and biotic stress in plants [11]. This has revealed the role of SNP as an exogenous NO donor in alleviating abiotic stresses, particularly those caused by heavy metals [2,12,19,20,21,22]. Nitric oxide has been observed to both suppress and induce cell death, depending on a variety of factors such as the dose and flux of local nitric oxide, plant species, and various concentrations of heavy metals. For instance, in cell suspension cultures of *ARABIDOPSIS,* grown at two different concentrations of CdCl_2_, nitric oxide with accelerated senescence produced cell death [23]. On the other hand, NO can help to mitigate and detoxify the high concentrations of ROS that cause detrimental effects on plant cells [24]. The protective role of NO in reducing ROS in plants has been indicated in several studies [13,25]. However, the positive action of nitric oxide depends on the flux and concentrations of nitric oxide and heavy metals, plant species, and nitric oxide quantification methods [26,27]. Numerous studies have reported that SNP contributes to the preservation of plant cells during stressful conditions [28,29,30]. However, there is not enough knowledge regarding the effects of different SNP doses as a nitric oxide donor on heavy metals. In this attempt, we will explore the role of high and low SNP concentrations in the reduction of toxicity in *Pleioblastus pygmaea* L. in order to determine the optimal SNP detoxification levels. To the best of the authors’ knowledge, this is the first study in this field on bamboo species (*Pleioblastus pygmaea* L.). We hypothesize that increasing the antioxidant capacity of SNP alleviates oxidative stress and reduces ROS compounds in plants, and that high concentrations of SNP have a significant effect on the reduction of H_2_O_2_ content and ROS compounds. Bamboo plants belong to the *Poaceae* family, which is classified as *Bambusoideas* in a subfamily [31,32]. Every year, new bamboo shoots emerge from the bamboo rhizomes in bud sites and bamboo shoots expand into a new culm and emerge in spring, while bamboo rhizomes and their root systems expand during the year. Bamboo growth increases in summer and autumn [33]. Bamboo, as a perennial evergreen woody plant, is known to be a cost-effective forestry product [34,35]. It is widespread across the tropics and subtropics and covers a large area (>6 million hectares) of Chinese forestland. Bamboo plants have been categorized into 500 species and 70 genera [36,37]. Bamboo’s rapid growth and huge biomass make it an excellent candidate for pollutant bioaccumulation as well as plant phytoremediation purposes [33]. In addition, Bamboo leaves are known as an effective tool for the removal of pollution in wastewater [38]. On the other hand, this remarkable plant, as an Asia native, is one of the primary economic resources and livelihoods of indigenous people in the southern and southeast regions of Asia [33]. It contributes significantly to the local populace’s economy, with over US $19.7 billion granted by the State Forestry Administration of China Beijing, China, 2012 [39]. Some bamboo species as ornamental plants have been used as eco-friendly and sustainable solutions to remove contamination and clean up air pollution in urban areas, thereby enhancing the commercial aspect of gardening and beautification by serving as a pollution indicator for phytoremediation purposes [40]. *Pleioblastus pygmaea* L. has been used in numerous landscaping projects as an ornamental and evergreen bamboo species. This bamboo species (which grows to a height of 30–50 cm height) was introduced into China from Japan in the last few decades. It has been known as a resistant bamboo in a variety of soil types, including neutral, basic (alkaline), and acidic soils [41]. Additionally, in some Chinese provinces, such as Jiangsu, it has been employed for urban beautification, and additionally for landscape purposes. *Pleioblastus pygmaea* L. has been identified as a viable plant for soil, air, and environmental pollution clearance. As a result of growing anthropogenic activity and industrialization in recent decades, a huge portion of China’s agricultural and forest land has become contaminated with toxic metals such as Mn and Cr, posing a serious threat to human health [42]. Southeastern China is the largest bamboo shoot-producing region, in recent research on six bamboo species in this region, Mn and Cr are introduced as two main polluting metals which can threaten the human food chain with high accumulation in shoots [43]. Thus, it is essential to focus on identifying the most effective means for reducing pollution from soil and plants in this area, as well as investigating techniques to increase bamboo’s tolerance against metal toxicity. The primary objective of this research is to evaluate the possibility of increasing plant tolerance by stimulating involved mechanisms such as antioxidant activity and relative water content, as well as reducing heavy metal accumulation and limiting root to shoot translocation, using high and low doses of SNP as a nitric oxide donor to bamboo plants under Mn and Cr.

## 2. Results

### 2.1. Mn and Cr Accumulation in Root, Stem, and Leaves in Bamboo Species

Metal accumulation in various plant organs is one way for plants to respond to the increased stress caused by heavy metals. According to the findings of the present study, increasing the concentration of SNP significantly reduces the Mn and Cr accumulation in *Pleioblastus pygmaea* L. The results of this experiment revealed a significant difference between the various levels of SNP alone and in combination with Mn and Cr (*p* < 0.001). Thus, the greatest reduction in Mn and Cr was attributed to a combination of 400 µM SNP, 150 µM Mn, and 150 µM Cr, which resulted in 55 and 48% reductions in bamboo leaves, 61 and 48% reductions in bamboo stems, and 59 and 50% decrements in bamboo roots, respectively, in comparison to their control. On the other hand, as reported in Table 1, the content of heavy metals (i.e., Mn and Cr) in roots is greater than that in stems and leaves, demonstrating the role of SNP in reducing metal translocation from roots to stems and leaves. As a result, we hypothesized that SNP as a nitric oxide (NO) donor has the potential to significantly reduce metal accumulation in plant organs such as roots, stems, and leaves, which could be due to nitric oxide’s ability to absorb and bind Mn and Cr ions, or it could be due to nitric oxide’s role as a physical barrier to metal translocation to stems and leaves. In any case, SNP as a nitric oxide (NO) donor increases the uptake of nutrient elements while reducing the absorbance of toxic metals such as Mn and Cr in *Pleioblastus pygmaea* L. (Table 1).

### 2.2. Tocopherols, Flavonols, and Total Phenolics in Plants under Mn and Cr

**Figure 1:**
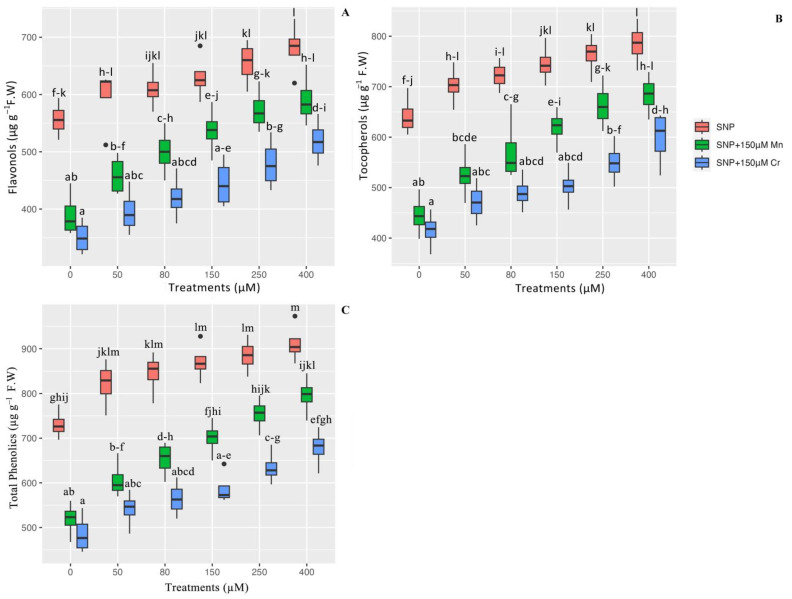
The effect of various concentrations of SNP individually and in combination with Mn and Cr on non-enzyme antioxidant activity (flavonols (**A**), tocopherols (**B**), and total phenolics (**C**)). The data indicated the mean ± standard error of four repetitions. The treatments include five different concentrations of SNP, either individually or in combination with 150 μM manganese and 150 μM chromium. Different lower-case letters indicate significant differences across all treatments based on Tukey′s test (*p* < 0.05).

The levels of flavonols, tocopherols, and total phenolics were measured to ascertain the effect of SNP on the stimulation of plants’ non-antioxidant activity in the presence of Mn and Cr toxicity. Our results indicate that there is a significant difference in the indices of flavonols, tocopherols, and total phenolics between the different concentrations of SNP in a combination of Mn and Cr (*p* < 0.001), which has demonstrated that SNP concentrations significantly increase flavonols, tocopherols, and total phenolics. Thus, the greatest enhancement was associated with 400 µM and 250 µM SNP concentrations, which increased flavonols by 22% and 19%, tocopherol by 22% and 18% and total phenolics by 24% and 21%, respectively, in comparison to their control treatments. On the other hand, the results indicated that 400 µM SNP in a combination of Mn and Cr enhanced non-antioxidant activity the most, with 51% and 47% increases in flavonols, 53% and 44% increases in tocopherols, and 53% and 39% increases in total phenolics, respectively, when compared to their control treatments. We hypothesized that varying the concentration of SNP enhances non-antioxidant activity as a second metabolism in *Pleioblastus pygmaea* L. (Figure 1).

### 2.3. Proline Contents (Pro), Glycine Betaine (GB), Glutathione (GSH), and Relative Water Content (RWC) in Pleioblastus pygmaea L. under Mn and Cr

**Figure 2:**
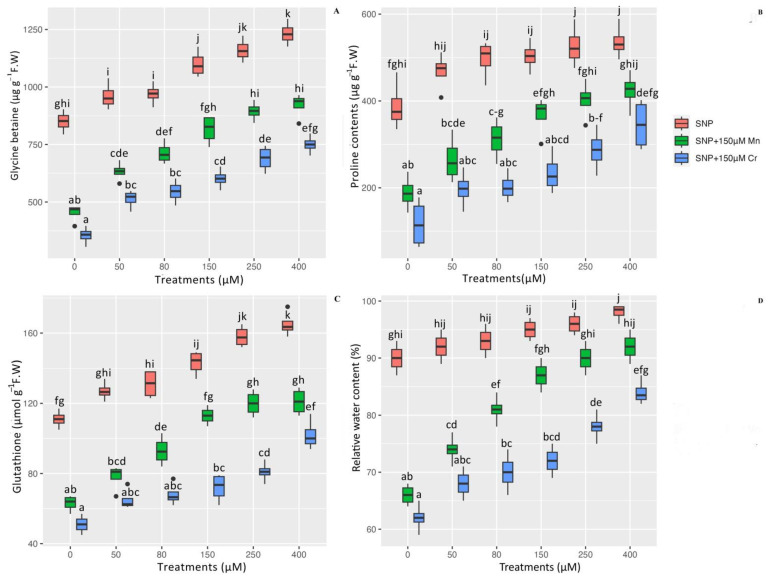
The effect of various concentrations of SNP individually and in combination with Mn and Cr on glycine betaine (GB) (**A**), proline content (Pro) (**B**), glutathione (GSH) (**C**), and relative water content (RWC) (**D**) in *Pleioblastus pygmaea* L. exposed to Mn and Cr. The data indicated the mean ± standard error of four repetitions. The treatments include five different concentrations of SNP, either individually or in combination with 150 μM manganese and 150 μM chromium. Different lower-case letters indicate significant differences between treatments as determined by Tukey′s test (*p* < 0.05).

To determine the efficacy of SNP in reducing heavy metal toxicity, several important indices such as the content of GB, Pro, GSH, and RWC were measured. According to the data analysis, there is a significant difference in the effects of SNP in combination with Mn and Cr on GB, Pro, GSH, and RWC (*p* < 0.001). While the 150 µM Mn and 150 µM Cr reduced the content of GB, Pro, GSH, and RWC in *Pleioblastus pygmaea* L, the addition of various concentrations of SNP significantly increased the content of GB, Pro, GSH, and RWC in *Pleioblastus pygmaea* L. exposed to Mn and Cr. According to the results, the greatest increase in the content of these indices was attributed to high concentrations of SNP (400 µM and 250 µM), which increased GB by 44% and 36%, proline (Pro) by 38% and 35%, GSH by 48% and 42%, and RWC by 8% and 6%, respectively. On the other hand, the results indicated that 150 µM Mn and 150 µM Cr had the greatest reductions in the content of GB, Pro, GSH, and RWC, with 46%, 51%, 43%, and 26% reductions for 150 µM Mn and 58%, 69%, 54%, and 31% reductions for 150 µM Cr, respectively, in comparison to their control treatment. We can conclude that all concentrations of SNP significantly increased the content of GB, Pro, GSH, and RWC in plants exposed to Mn and Cr. However, the highest concentrations of SNP were more effective at increasing these indices (Figure 2).

### 2.4. ROS Component and Amelioration Lipid Peroxidation in Pleioblastus pygmaea L. under Mn and Cr Stress

**Figure 3:**
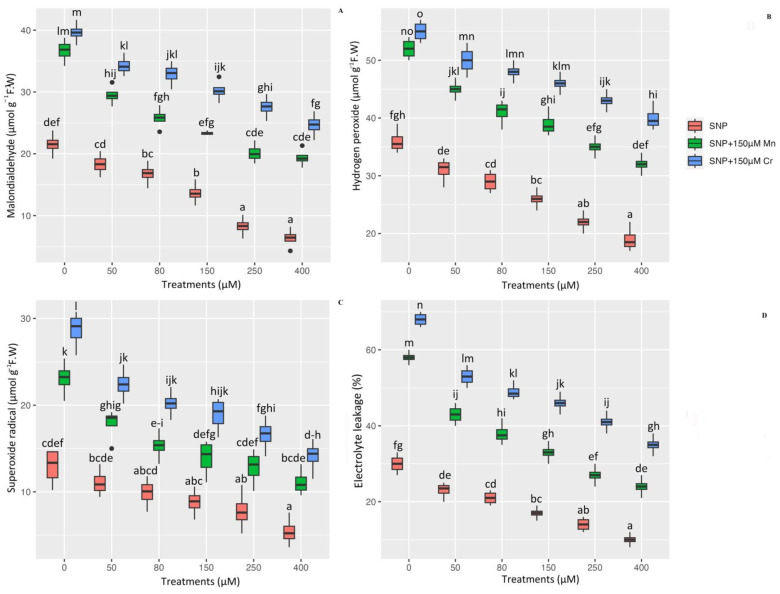
The effect of various concentrations of SNP individually and in combination with Mn and Cr on malondialdehyde (MDA) content (**A**), hydrogen peroxide (H_2_O_2_) (**B**), superoxide radical (O_2_^•−^) (**C**), and electrolyte leakage (EL) (**D**) in *Pleioblastus pygmaea* L. exposed to Mn and Cr. The data indicated the mean ± standard error of four repetitions. The treatments include five different concentrations of SNP, either individually or in combination with 150 μM manganese and 150 μM chromium. Different lower-case letters indicate significant differences between different concentrations of SNP used individually or in combination with 150 μM Mn and 150 μM Cr, as determined by Tukey′s test (*p* < 0.05).

The investigation of the detrimental effects of metal toxicity on the generation of ROS compounds and plant cell membranes is a critical issue in plants under stress. As a result, it is necessary to determine the ROS compounds, such as H_2_O_2_ and O_2_^•−^ as well as lipid peroxidation indicators, such as MDA and electrolyte leakage, in plants under stress. The results of this study revealed a significant difference in the indices of MDA, H_2_O_2_, EL, and O_2_^•−^ between different concentrations of SNP with 150 µM Mn and 150 µM Cr (*p* < 0.001). Thus, while 150 µM Mn and 150 µM Cr increased cell oxidation and lipid peroxidation, SNP significantly reduced MDA, H_2_O_2_, O_2_^•−^, and EL in *Pleioblastus pygmaea* L. exposed to Mn and Cr, respectively. This result showed the greatest reduction was the high concentration of SNP (400 µM SNP), which reduced MDA, H_2_O_2_, O_2_^•−^, and EL by 70%, 47%, 58%, and 66%, respectively, in comparison to their control treatment. On the other hand, the results revealed that the combination form of 400 µM SNP with heavy metals (150 µM Mn and 150 µM Cr) resulted in the greatest reduction in the indices of Malondialdehyde (MDA), hydrogen peroxide (H_2_O_2_), superoxide radical (O_2_^•−^), and electrolyte leakage (EL) with enhancements of 70%, 44%, 79%, and 93% by 150 µM Mn and 84%, 52%, 96%, and 99% by 150 µM Cr, respectively, in comparison to their control treatments. We hypothesized that different concentrations of SNP mitigated the detrimental effects of ROS components (H_2_O_2_ and O_2_^•−^) on plant cells and protected the cell membrane from oxidation, resulting in a decrease in MDA content and EL percentage (Figure 3).

### 2.5. Antioxidant Enzyme Activity in Plants Exposed to Mn and Cr Toxicity

**Figure 4:**
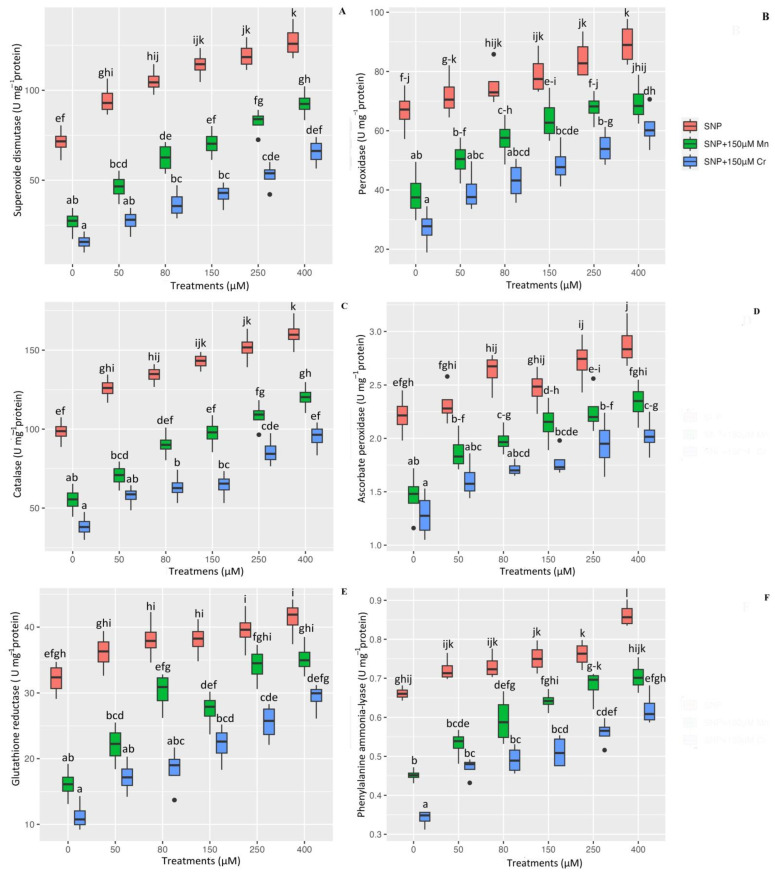
The effect of various concentrations of SNP individually or in combination with Mn and Cr on antioxidant enzyme activity, such as superoxide dismutase (SOD) (**A**), peroxidase (POX) (**B**), catalase (CAT) (**C**), ascorbate peroxidase (APX) (**D**), glutathione reductase (GR) (**E**), and phenylalanine ammonia-lyase (PAL) (**F**) in *Pleioblastus pygmaea* L. exposed to Mn and Cr. The data indicated the mean ± standard error of four repetitions. The treatments include five different concentrations of SNP, either individually or in combination with 150 μM manganese and 150 μM chromium. Different lower-case letters indicate significant differences between different concentrations of SNP used individually or in combination with 150 μM Mn and 150 μM Cr based on Tukey′s test (*p* < 0.05).

Antioxidants are the first line of plant defense for plants exposed to heavy metal stress. They play a critical role in protecting plant cells from ROS compounds and free radicals, as well as preventing plant oxidation under metal stress. The present study’s data analysis revealed a significant difference in the activity of antioxidant enzymes such as SOD, POX, CAT, APX, GR, and PAL between the different concentrations of nitric oxide and 150 µM Mn and 150 µM Cr (*p* < 0.001), which showed that various concentrations of SNP significantly increased antioxidant enzyme activity. However, the results indicated that the highest concentration of nitric oxide (400 µM) is the most effective at enhancing plant antioxidant activity under stress, with increases in SOD, POD, CAT, APX, GR, and PAL activity of 78%, 34%, 62%, 30%, 28%, and 24%, respectively, when compared to the control treatments. Additionally, the results indicated that Mn and Cr had the lowest increase in antioxidant capacity, with 150 µM Mn and 150 µM Cr increasing SOD by 62% and 77%, POD by 44% and 59%, CAT by 43% and 61%, APX by 33% and 42%, GR by 49% and 64%, and PAL by 31% and 48%, respectively, when compared to control treatments. We can suggest that all concentrations of SNP increase the antioxidant activity of Mn and Cr in plants, thereby protecting cells from oxidation. However, the results indicated that as SNP levels increased, the antioxidant capacity increased as well (Figure 4).

### 2.6. Plant Photosynthetic Pigments, including Chlorophyll a, Chlorophyll b, Total Chlorophyll and Carotenoids Exposed to Mn and Cr

Photosynthesis parameters are critical for evaluating plant metabolism under stress conditions that can directly affect plant growth and development. For this purpose, we measured photosynthetic pigments, including chlorophyll *a* and chlorophyll *b* as well as carotenoids, in *Pleioblastus pygmaea* L. under Mn and Cr with and without the addition of various levels of SNP. We hypothesized that the SNP could enhance photosynthesis properties. The results indicated that there is a significant difference between the various levels of SNP when combined with Mn and Cr (*p* < 0.001). As a result, we discovered that increasing SNP levels increased photosynthetic pigments in plants exposed to metal toxicity. However, the greatest increase in chlorophyll *a*, chlorophyll *b*, total chlorophyll, and carotenoids was associated with 400 µM and 250 µM SNPs, with increases of 13% and 10% in chlorophyll *a*, 35% and 30% in chlorophyll *b*, 39%, and 33% in total chlorophyll, and 34% and 29% in carotenoid, respectively, in comparison to their control treatment. On the other hand, 400 µM SNP had the greatest effect on increasing photosynthesis parameters in plants grown in 150 µM Mn and 150 µM Cr, with 57% and 58% increases in chlorophyll-*a*, 90% and 74% increases in chlorophyll-*b*, 72% and 65% increases in total chlorophyll, and 70% and 95% increases in carotenoid content, respectively, in comparison to their control treatments. Samples treated with 150 µM Mn and 150 µM Cr reduce photosynthesis pigments by 35% and 41% in chlorophyll-*a*, 41% and 48% in chlorophyll-*b*, 30%, and 37% in total chlorophyll, and 30% and 50% in carotenoids, respectively, in comparison to control treatment (Table 2).

### 2.7. Plant Growth, and Plant Biomass on the Dry Weight of Shoot and Root as Well as Plant Shoot Length under Mn and Cr

**Figure 5:**
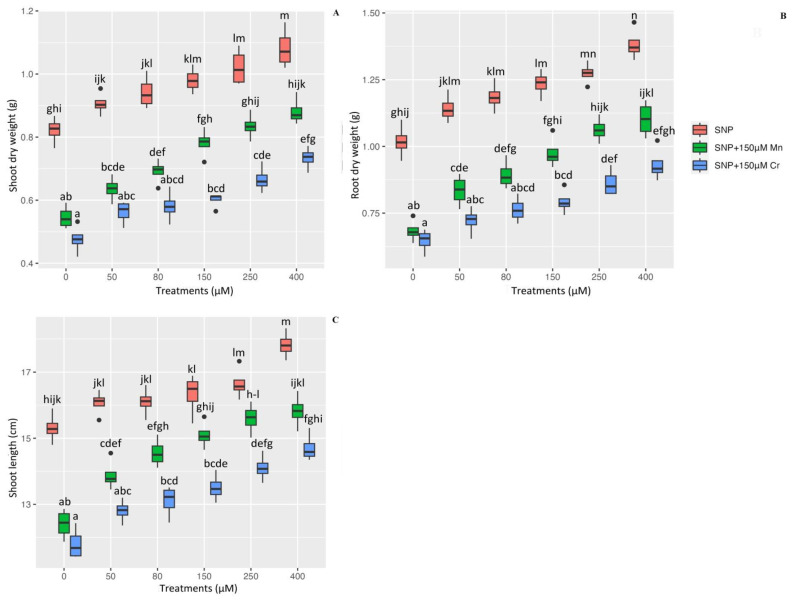
The effect of various concentrations of SNP individually and in combination with Mn and Cr on shoot dry weight (**A**), root dry weight (**B**), and shoot length (**C**) in bamboo species exposed to Mn and Cr. The data indicated the mean ± standard error of four repetitions. The treatments include five different concentrations of SNP, either individually or in combination with 150 μM manganese and 150 μM chromium. Different lower-case letters indicate significant differences between different concentrations of SNP used individually or in combination with 150 μM Mn and 150 μM Cr based on Tukey′s test (*p* < 0.05).

Shoot and root dry weights, as well as shoot length, are used as indicators of plant biomass and growth in this study, which are necessary to demonstrate the morphologic impact of SNP on heavy metal toxicity. According to the results, there was a significant difference in shoot and root dry weight, as well as plant shoot length, between the different levels of SNP in combination with 150 µM Mn and 150 µM Cr (*p* < 0.001) (Figure 5). This indicated that various concentrations of SNP enhance plant growth and biomass indexes both individually and in combination with Mn and Cr. However, the greatest increase was observed with 400 µM and 250 µM SNP, respectively, with 1.08 g and 1.02 g shoot dry weight, 1.38 g and 1.27 g root dry weight, and 17.02 cm and 16.65 cm in shoot length, respectively. Additionally, the results revealed that 400 µM SNP in plants under Mn and Cr increases plant biomass by 61% and 54% in shoot dry weight, 61% and 44% in root dry weight, and 27% and 24% in shoot length, respectively, in comparison to their control treatment. This demonstrated that varying concentrations of SNP mitigated the destructive effects of heavy metals on plant growth. According to Table 3, 150 µM Mn and 150 µM Cr significantly reduce shoot dry weight by 33% and 42%, root dry weight by 32% and 36%, and plant shoot length by 18% and 22%, respectively when compared to the control treatment (Table 3). We hypothesized that varying levels of SNP increase plant biomass as well as plant growth exposed to Mn and Cr, which is associated with an increase in *Pleioblastus pygmaea* L. antioxidant capacity. This in turn increases chlorophyll pigments and, ultimately, plants’ growth.

### 2.8. Effects of Nitric Oxide on Plant Tolerance Indices (TI) in Shoot and Root, as Well as Bio Accumulation Factor (BAF) and Plant Translocation Factor (TF) in Pleioblastus pygmaea L. Exposed to Mn and Cr Toxicity

The TI, BAF, and TF all play critical roles in determining a plant’s resistance to metal toxicity. In fact, these are some of the major mechanisms by which nitric oxide can reduce metal toxicity in plants. Our findings indicate that there is a statistically significant difference between various levels of SNP in combination with Mn and Cr in TI, BAF, and TF (*p* < 0.001) (Table 4). Thus, the results indicate that TF from root to leaf decreases with the addition of SNP, with the greatest reduction attributed to the combination of high concentrations of SNP with Mn and Cr, which resulted in a 9% and 8% decrease in the translocation factor of Mn and Cr, respectively. Additionally, the results indicated that BAF of Mn and Cr decreased by 56% and 49% in the leaves, respectively, when compared to the control treatment. However, the BAF in the leaves and stem was less than that in the roots, indicating that nitric oxide helps to accumulate Mn and Cr in roots, thereby limiting metal toxicity transfer to the stem and leaves, potentially improving the tolerance factor in *Pleioblastus pygmaea* L. Our results demonstrate that the 400 µM and 250 µM SNP concentrations have the highest TI in the bamboo species, increasing the TI of the shoot by 31% and 24%, and the TI of the root by 35% and 25%, respectively, when compared to the control treatment. On the other hand, evaluation of the various levels of SNP indicated that nitric oxide has the potential to increase TI in *Pleioblastus pygmaea* L. exposed to Mn and Cr; thus, the greatest increase in TI in shoot and root was attributed to the combination of 400 µM SNP with 150 µM Mn and 150 µM Cr, which resulted in a 61% and 54% increase in shoot tolerance index and a 61% and 43% increase in root tolerance index, respectively, when compared to control treatments. According to Table 1, roots accumulate more Mn and Cr than stems and leaves, indicating that bamboo roots are capable of accumulating a significant amount of Mn and Cr. This is consistent with previous research indicating that bamboo accumulates in tissues in the rhizome and culm, primarily in the radius of the vacuole, cell wall, and cytoplasm of a plant cell. As a result, we concluded that SNP enhances the phytoremediation potential of *Pleioblastus pygmaea* L. in polluted areas by increasing the accumulation of Mn and Cr on the root surface.

## 3. Discussion

Nitric oxide is a gaseous molecule with multifunctions (lipophilic, paramagnetic gas, free radical, and charge-free with a short half-life) that has the ability to mitigate the toxicity of heavy metals at both exogenous and endogenous levels [44,45,46]. It has been implicated as a NO-mediated mediator in a variety of physiological processes in plants exposed to different types of stresses, including heavy metals [47]. Nitric oxide has two significant mechanisms by which it can alleviate oxidative stress in plants. Firstly, nitric oxide acts directly as a scavenger of ROS compounds, forming peroxynitrite when it reacts with superoxide radicals. Therefore, nitric oxide reduces cell toxicity, resulting in less cell damage in plants [48]. The second action is related to the signaling role of nitric oxide in the reduction of stress, as nitric oxide is a signaling molecule involved in antioxidative gene expression alterations [49]. On the other hand, in plants under stress, ROS regulates electron transport pathways in plant organs and tissues such as mitochondria. Where ROS modulation occurs, it stimulates the plant’s defense mechanism, resulting in an increase in the activity of antioxidant enzymes [50]. Exogenous nitric oxide with activating antioxidant capacity scavenges free radicals such as organic radicals, superoxide anion, and lipid O_2_ as well as H_2_O_2_ [51,52]. Numerous studies have demonstrated that nitric oxide increases antioxidant activity in plants exposed to heavy metals [53,54,55,56,57], which was also observed in the present study. Thus, in order to determine whether the decrease in Mn and Cr toxicity in bamboo plants is related to the antioxidant capacity of nitric oxide, the activity of antioxidant enzymes such as SOD, POD, CAT, APX, GR, and PAL was examined. Our results demonstrated that increasing the concentration of SNP as one nitric oxide donor increased antioxidant enzyme activity, which is consistent with the activation of the antioxidant enzyme’s gene expression induced by high and low SNP doses. Flavonols, tocopherols, and total phenolics are all important non-enzyme antioxidants that protect against heavy metal toxicity. In fact, increasing flavonols and total phenolics is a critical strategy for plants exposed to heavy metals [58,59]. Due to the electron-donating agents, phenolic compounds act as antioxidants, scavenging ROS compounds and free radicals [60,61]. Additionally, flavonols have antioxidant properties in abiotic stores [62]. When a plant is stressed, it accumulates more flavonols to protect itself [63]. It appears that SNP as a nitric oxide donor stimulates gene expression involved in flavonoid biosynthesis, thereby increasing flavonoid accumulation in plants exposed to metal toxicity [64]. Our data analysis revealed that varying SNP concentrations can enhance non-antioxidant activity in *Pleioblastus pygmaea* L. exposed to Mn and Cr. As a result, we hypothesized that SNP, through its effects on gene expression involved in the biosynthesis of non-antioxidant enzyme activity (flavonols, tocopherols, and total phenolics), may enhance plant second metabolism under stress conditions.

MDA is one indicator of increased lipid peroxidation in plants exposed to excessive amounts of heavy metals [65]. H_2_O_2_ is generated in plant cells in response to metal toxicity [66]. Exogenous nitric oxide has been shown to reduce MDA levels as well as ROS compounds in a variety of plant species, including rice and mung bean [44,45]. On the other hand, nitric oxide has the potential to directly eliminate the toxic effects of O_2_^•−^ through the conversion of O_2_^•−^ to ONOO− [67]. As we hypothesized in our current study, the addition of SNP as a nitric oxide donor to Mn and Cr reduces the superoxide radical (O_2_^•−^) content. Our findings indicated that 150 µM Mn and 150 µM Cr increased H_2_O_2_, resulting in increased lipid peroxidation and electronic leakage. Our findings, however, indicate that the addition of SNP remarkably reduces Mn and Cr toxicity, thereby limiting lipid peroxidation and electrolyte leakage across the plant cell membrane. Thus, we hypothesized that increased antioxidant activity of SNP scavenges ROS compounds such as H_2_O_2_ and O_2_^•−^, which contribute to increased lipid peroxidation and electrolyte leakage across the cell membrane. Methylglyoxal (MG) is a component of a reactive cytotoxic α-oxo aldehyde that is abundantly produced in stressed plants [68,69]. Stimulating GSH synthesis via nitric oxide levels is one of the primary defense mechanisms against plant cell oxidation [70], which is associated with the GSH-mediated regulation of MG levels [71,72,73]. Thus, Gly I, the first enzyme in the glyoxalase pathway, was co-factored by GSH during MG reduction, and nitric oxide can impact the glyoxalase pathway enzymes [74]. Our findings indicated that increasing the concentration of SNP significantly increased the GSH concentration in plants exposed to Mn and Cr, demonstrating the protective role of nitric oxide in a plant cell.

By accumulating osmotic constituents, nitric oxide preserves cell water content and thus alleviates plant stress [75]. Among osmolytes, glycine betaine and proline have beneficial effects on membrane stability, plant osmoregulation, and plant stress reduction. Glycine betaine and proline have been shown to act as co-factors in the activity of hydrated enzymes [76]. Glycine betaine is a major compatible substance that has been shown to regulate plant osmotic balance under stress conditions [77]. Additionally, it has been reported that glycine betaine plays a beneficial role in increasing photosynthetic pigments. Thus, accumulation of glycine betaine can improve photosynthesis efficiency in plants that are exposed to heavy metals [78]. Proline can either fix protein complexes in plant cells or directly scavenge oxygen free radicals. Additionally, proline acts as a signal for downstream events to occur [79]. SNPs have been shown to increase proline levels in stressed plants. The correlation between proline and nitric oxide is due to the fact that they both utilize L-arginine as a common precursor in their biosynthesis [56]. On the other hand, numerous studies have reported an increase in the glycine betaine and proline levels in plants exposed to heavy metals [80,81,82]. Our results establish a correlation between proline and glycine betaine levels, demonstrating that when nitric oxide is added, the content of proline and glycine betaine accumulates in *Pleioblastus pygmaea* L. exposed to Mn and Cr toxicity. The RWC of the leaves is a critical factor in the reduction of heavy metal stress. Nitric oxide increases water content by increasing wall extensibility, cell division, and plant morphological characteristics such as leaf area, weight, and length [82]. While 150 µM Mn and 150 µM Cr decreased the RWC of *Pleioblastus pygmaea* L., the addition of SNP increased the index of RWC in *Pleioblastus pygmaea* L. exposed to toxicity. This has already been demonstrated in numerous publications on bean seeds [45] and wheat (*Triticum aestivum* L.) [56,83].

The accumulation of heavy metals in plants reduces the synthesis of chlorophyll pigments, which is related to changes in the chlorophyll biosynthetic intermediates, and thus has a detrimental effect on the pigment–protein complex [84]. Nitric oxide preserves the chlorophyll pigment and chloroplast membrane through the viscosity of the cytoplasm, which is maintained through the preservation of osmotic pressure [82]. Numerous studies have documented the role of nitric oxide in alleviating the negative effects of heavy metals on photosynthesis as well as promoting photosynthesis properties [46,55,85,86,87]. This is consistent with our findings in *Pleioblastus pygmaea* L, indicating that different concentrations of SNP significantly increase photosynthesis pigments such as chl-*a*, chl-*b*, total chl, and carotenoid in plants grown in 150 µM Mn and 150 µM Cr. Nitric oxide has been shown to regulate plant abiotic stress tolerance through alteration of exogenous nitric oxide levels, resulting in increased crop and plant production under stressful conditions [82,88]. *Typha angustifolia* under cadmium [80], *vicia faba* under arsenic [89], and *oryza sativa* (rice) under cadmium [90] demonstrated the effect of nitric oxide on promoting plant biomass and growth. This can be explained by nitric oxide’s inducing effect on cell wall relaxation and expansion, as well as its protective effect on the phospholipid bilayer, which results in increased plant growth and development under stress [91,92]. On the other hand, it has elucidated the role of nitric oxide in the protective alteration of plant roots against oxidative damage in several plant species, including *Citrus grandis* [93], *Oryza sativa* [44,94], *Brassica juncea* [46], and *Lupinus luteus* [95], which can be an important issue in improving plant growth. Additionally, there is another reason that demonstrates the efficiency of nitric oxide in plant growth. That is attributed to the role of nitric oxide in enhancing the osmotic pressure within the cell as well as ameliorating the viscosity of the cytoplasm [96,97]. As a result, it can be concluded that SNP as a nitric oxide donor has the ability to increase plant growth and biomass in the presence of heavy metals, as demonstrated in our study. Thus, our study revealed that various concentrations of SNP increase plant biomass and growth (dry weight of shoot and root as well as shoot length) in the presence of Mn and Cr. This has been demonstrated by the beneficial effects of Mn on *Oryza sativa* (rice) growth [98] and Cr on *triticum aestivum* (wheat) growth [80]. Additionally, our results indicate that 400 µM SNP has the greatest effect on plant growth and biomass, implying that a high concentration of SNP could be significantly more effective at promoting plant growth in the presence of Mn and Cr toxicity.

It has been reported that the initial mechanisms of the protective role of nitric oxide in various doses and forms are likely to begin with the reduction of metal accumulation [99] and progress to the amelioration of metal-induced oxidative stress [100,101]. The findings of this study indicate that SNP as a nitric oxide donor limits the accumulation and uptake of heavy metals in plant organs, which can be a protective mechanism against metal stress, and this has been confirmed by other studies as well [82,102]. On the other hand, in one study on rice, nitric oxide resulted in the enhancement of hemicellulose and pectin, which could improve Cd detoxification by increasing the accumulation of Cd in the root cell walls while decreasing the Cd content in the leaves’ cell walls [103]. This is consistent with our findings in the current study. As a result, the findings revealed that metal accumulation in plant roots is greater than that in the shoot and stem, demonstrating the beneficial effect of SNP on the reduction of heavy metal translocation from root to shoot. Hence, SNP can absorb and bind with metal ions on the root surface, preventing the transfer of Mn and Cr from the root to the aerial parts, thereby increasing the tolerance factor of the plant under stress. Additionally, it has been suggested that the combination of SNP and heavy metal accumulation on the root surface and root cell walls increases plant phytoremediation and plant recovery. 

## 4. Materials and Methods

### 4.1. Experiment Materials and Vitro Plant Tissue Culture

**Figure 6:**
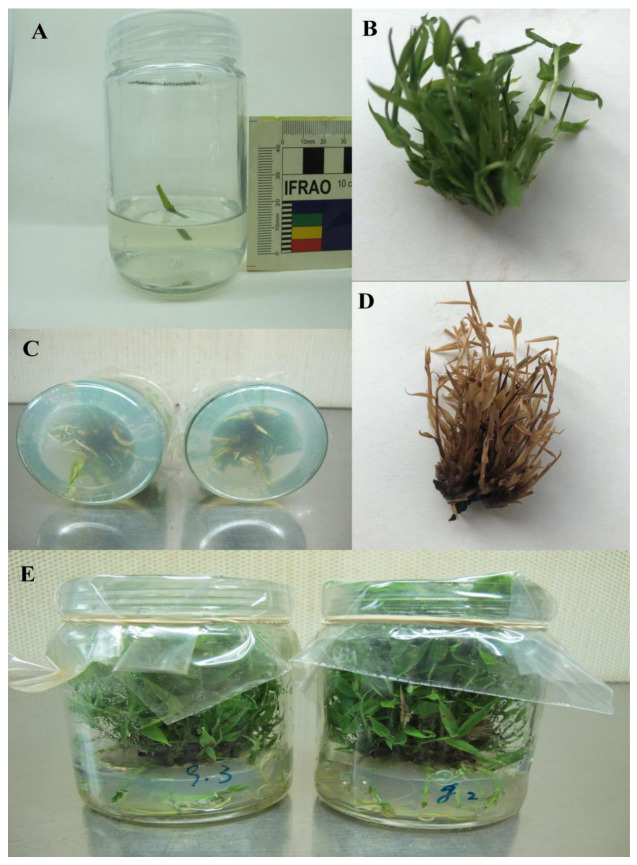
Phenotypes of bamboo plants in vitro condition (**A**), 10 mm long nodal explants of *P. pygmaeus* treatments (**B**), control treatment of bamboo species (**C**), roots proliferation from bamboo shoots (**D**), bamboo treatment exposed to 150 µM Cr (**E**), bamboo treatments in plant tissue culture room chamber.

A single clone of *P. pygmaeus*, a one-year-old sapling, was provided by the bamboo research base garden at Nanjing Forestry University. For the pre-experimental study, approximately 10 mm-long nodal explants of *P. pygmaeus* treatments were used to initiate this experiment. The MS medium [104] was employed in the tissue culture of nodal explants in a vitro environment. It comprised of 4 μM 6-benzyl amino purine (6-BAP), 0.5 µM kinetin (KT), 25 g/L sucrose, and 7–10 g/L agar and the explant was left for two weeks. To promote root proliferation, shoots were transferred and cultured in an MS medium containing 1.2 μM thiamine –HCl, 4 μM nicotinic acid, 0.6 mM myo-inositol, and 3 μM pyridoxine, as well as 150 μM Mn and 150 μM Cr alone or in combination with various concentrations of SNP added to 30 g/L sucrose, 8–10 g/L agar, and 0.1 mg/L indole-3-acetic acid (IAA) (growth hormone regulator) at pH 5.8 ± 0.1. Thus, one liter of the prepared MS medium was placed in an oven (HiClave HVE-50) and sterilized for 30 min at 120–130 °C. After sterilizing, the medium was allowed to cool to room temperature before each treatment of bamboo species was cultured in a glass petri dish measuring 60 and 90 mm in diameter and height, respectively, and containing 100 mL of plant culture medium in an air tech ultraviolet-sterilized incubation hood. This lasted for five hours under white fluorescent light with a wavelength range of 20–410 nm at a temperature of 20 °C. The treatment of bamboo was then transferred to a single plant tissue culture room chamber for three weeks under controlled conditions for a 15-h photoperiod with light and dark phases of 28–24 °C and 18–23 °C, respectively (Table 5) (Figure 6).

### 4.2. Estimation of Metal Content and Nitric Oxide Accumulation in Roots, Stems, and Leaves

To determine the Mn and Cr content of root stems and leaves, 0.5 g of plant samples was added to a solution containing 2 mL of HNO_3_ (67% *w*/*v*) and 2 mL of H_2_O_2_ (30% *v*/*v*). The resulting solution was then added to a mixture containing 10% (*v*/*v*) HCl, 10% KI (*w*/*v*), and 5% ascorbic acid (*w*/*v*). In the final step, a Shimadzu AA-6200 atomic absorption spectrometer (HG-AAS), was utilized to determine the accumulation of Mn and Cr using an external standard [105]. The accumulation of nitric oxide was detected by converting the oxygen–hemoglobin content to methemoglobin. Thus, in this approach, 0.5 g of root, stem, and leaf samples were added to a mixture containing sodium acetate (0.1 M), 3 mL of buffer (pH 6.0), 1 M NaCl, and 1% (*w*/*v*) ascorbic acid. It was then centrifuged for 25 minutes at 7000× *g* at 5 °C [106,107].

### 4.3. Determination of Tocopherols, Flavonols, and Total Phenolics as Non-Antioxidant Activity

To prepare the methanolic extract, 0.5 g of samples (dry leaf) were added to 4 mL of methanol (80%) and centrifuged at 6000× *g* for 20 min. The Conde method was used to determine the total phenolics [108]. A folin–ciocalteu reagent (10% *v*/*v*) (2.5 mL) was added to 0.1 M of methanolic extract. Subsequently, 7% (*w*/*v*) sodium bicarbonate was added to the mixture to neutralize the soluble phase. Finally, to estimate the total phenolics in the combination, its absorbance at 765 nm was assessed using a spectrometer (Beijing Purkinje TU-1810UV-vis spectrometer, Beijing, China). The flavonol content was measured using the Akkol technique [109]. To this end, 0.4 mL of aluminum chloride (2% *w*/*v*) and 1.5 mL of sodium acetate (5% *w*/*v*) were added to 0.5 mL of methanolic extract. The obtained supernatant was then kept at the room temperature for three hours. The content of flavonols was determined using a single spectrometer by measuring the absorbance of the supernatant at 445 nm. Tocopherol was determined in this investigation using a protocol developed by Kayden [110]. In this method, 0.1 g of samples (leaves) were added to 3 mL of ethanol and centrifuged at 6000× *g* for 20 min before being added to a mixture containing 0.001 M of 0.2 mL of ferric chloride, 0.1 mL of ethanol extract, 0.2 mL of bathophenanthroline (0.2% *w*/*v*), and 1 mM of phosphoric acid (0.2 mL). The tocopherol content was determined by measuring the absorbance of the resulting supernatant at 534 nm.

### 4.4. Relative Water Content (RWC), Proline Content (Pro), Glutathione (GSH), and Glycine Betaine (GB)

Relative water content (RWC) analysis was conducted according to the protocol proposed by Barrs and Weatherly [111]. For this means, to determine the fresh weight (FW), the leaf lamina was weighed, and then the fresh leaves were floated on water in a petri dish for 10 h under dark conditions. Then, a paper towel was used to dry the surface water from the leaves and then the turgid weights (TW) were measured. To determine the dry weights (DW), leaves were dried for two days at 75 °C. The final RWC was calculated using the formula below:

RWC (%) = (FW − DW)/(TW − DW) × 100(1)

Glycine betaine (GB) was measured using the protocol proposed by Grieve and Grattan [112]. It was obtained by measuring the absorbance at 365 nm using a single standard curve. The proline content was determined by the protocol proposed by Bates [113]. To this end, sulfosalicylic acid was used to digest 330 mg of leaf samples, and supernatant absorbance at 520 nm was determined using a standard curve. Ellman’s methods [114] were used to determine the glutathione (GSH) content by recording an absorbance at 412 nm.

### 4.5. Lipid Peroxidation (MDA), Hydrogen Peroxide (H_2_O_2_), Electrolyte Leakage (EL), and Superoxide Radical (O_2_^•−^)

Malondialdehyde (MDA) was used as a lipid peroxidation biomarker in accordance with the Madhava Rao and Sresty’s methodology [115]. The MDA content was determined from spectrometer absorbance measurements at 600 and 532 nm. Hydrogen peroxide (H_2_O_2_) is a ROS compound that was obtained using the protocol proposed by Velikova et al. (2000) [116], with the final supernatant absorbance being measured at 390 nm using a spectrometer. The superoxide radical (O_2_^•−^) was determined using the protocol proposed by Li et al. (2010) [117]. It was obtained through the use of a nitrogen dioxide radical (NO2•) as a reference curve. Electrolyte leakage (EL) was obtained using the methods proposed by Valentovic et al. (2006) [118]. According to this method, 0.3 g of leaf samples were combined with 15 mL of deionized water. Next, the mixture was kept at a temperature of 20 °C for 3 h. The resulting solution was then used to determine the primary electrical conductivity (EC_1_). The samples were then placed in an autoclave set to 115 °C for 20 min. The resulting samples were then used to determine the secondary electrical conductivity (EC_2_). The final EC was calculated using the following formula:

EL (%)  =  (EC_1_/EC_2_)  ×  100 (2)

### 4.6. Antioxidant Activity

The 0.5 g of leaf samples were washed and cleaned before being cut into small species with scissors. The samples were then immersed in liquid nitrogen (LN) and crunched in a single mortar and pestle. The resulting powder was added to 4 mL of saline phosphate buffer (pH 7.2–7.4) at room temperature. Finally, the mixture was centrifuged at 3000–4000× *g* for 10 min to get the supernatant, which was used to estimate the antioxidant enzyme activity.

Superoxide dismutase (SOD) was measured using the protocol proposed by Zhang (1992) [119], which was based on the photo reduction of nitro blue tetrazolium (NBT). Peroxidase (POD) was estimated by measurement of the alteration in absorbance at 470 nm using the Zhang (1992) [119] method. Catalase (CAT) was measured using the Aebi et al. (1984) [120] method, which involved analyzing two H_2_O_2_ reactions at an absorbance of 240 nm. Glutathione reductase (GR) was determined using the protocol proposed by Foster and Hess (1980) [121], which used an absorbance of 340 nm. The ascorbate peroxidase (APX) was determined using the protocol proposed by Nakano and Asada (1981) [122] at 290 nm. The phenylalanine ammonia-lyase (PAL) activity was determined using the protocol proposed by Berner et al. (2006) [123].

### 4.7. Photosynthetic Pigments including Chlorophyll a, Chlorophyll b, Total Chlorophyll, and Carotenoids Content

Photosynthetic pigments contents, such as chlorophyll-*a*, chlorophyll-*b*, total chlorophyll, and content of carotenoids were estimated using the protocol proposed by Lichtenthaler and Buschmann (2001) [124]. According to this protocol, bamboo leaf samples weighing 0.5 g were crunched in an adequate amount of liquid nitrogen (LN). The resulting powder was homogenized in a single mortar and pestle in 20 mL of 80% (*v*/*v*) acetone at a temperature of 0 to 5 °C. The mixture treatments were then centrifuged at 7000× *g* for 15 min to determine the photosynthetic pigments. The resulting supernatant was transferred to a single spectrometer machine to determine chlorophyll *a*, and *b*, as well as carotenoid at absorbances of 663, 645, and 470 nm, respectively. The following formulae were used to determine the final content of photosynthetic pigments in mg/g F.W. (Fresh Weight) unit:

Total Chlorophyll = Chlorophyll *a* + Chlorophyll *b* Chlorophyll *a* = 12.25A663 − 2.79A647 Chlorophyll *b* = 21.50A647 − 5.10A663 Carotenoid = (1000A470 − 1.82Chl *a* − 95.15 Chl *b*)/225 (3)

### 4.8. The Calculation of Tolerance Index (TI) in shoot and Root, The Translocation Factor (TF) in Leaves and Stem, as Well as Bioaccumulation Factor (BAF) in Root, Stem, and Leaves

TF, TI, and BAF were calculated in this study using the method proposed by Souri and Karimi (2017) [125] according to phytoextraction efficiency, which is the primary indicator of plant phytoremediation.

The bioaccumulation factor (BAF) was calculated using the formula below:

BAF = (Concentrations of Mn and Cr in stem and shoot)/(Concentrations of Mn and Cr in the medium)(4)

The following formula was used to calculate the translocation factor (TF):

TF = (Concentrations of Mn and Cr in the shoot)/(Concentrations of Mn and Cr in the root)(5)

The tolerance index (TI) was determined using the formulae below:

TISH = (Shoot dry weight of Mn and Cr treatments)/(Shoot dry weight of control treatment)(6)

TIR = (Root dry weight of Mn and Cr treatment)/(Root dry weight of control treatment)(7)

### 4.9. Root Dry Weight, Shoot Dry Weight, and Shoot Length

After washing and cleaning the plant shoots and roots, they were placed in a vacuum drying oven (DZF-6090) at 115 °C for 20 min. Following that, treatments were dried in a fixed-dry weight mode at an oven temperature of 80 °C. Finally, dried samples were used as root dry weight DW and shoot dry weight DW, with each treatment repeated four times. To determine the shoot length, bamboo shoots were measured in two steps—one at the inception and the other at the end of the experiment.

### 4.10. Statistical Analysis

The research was conducted using a two-way factorial design with four repetitions using a completely randomized design (CRD). The data were analyzed using a statistical package for the analysis of variance (ANOVA) provided by R software. Tukey’s test was used to determine the mean difference between treatments at the probability level of *p* < 0.05.

## 5. Conclusions

This study implies that SNP as a signaling molecule could play a pivotal role in the amelioration of metal toxicity in bamboo species (*Pleioblastus pygmaea* L.), which is consistent with previous findings. While the addition of 150 um Mn and 150 um Cr increased oxidative stress in *Pleioblastus pygmaea* L., our data suggest that the addition of various levels of SNP as a nitric oxide donor improved plant tolerance and growth, as well as other toxicity. These improvements were achieved through enhancing antioxidant activity, regulating relative water content, reducing heavy metal accumulation, and restricting heavy metal translocation from root to shoot. According to the findings of the current study, increasing the concentrations of heavy metals results in an increase in: (1) Antioxidant activity such as superoxide dismutase (SOD), catalase (CAT), peroxidase (POD), glutathione reductase (GR), ascorbate peroxidase (APX), and phenylalanine ammonia-lyase (PAL); (2) non-antioxidant activity, such as tocopherols, flavonols, and total phenolics; (3) relative water content (RWC), proline contents (Pro), glycine betaine (GB), and glutathione (GSH); (4) photosynthesis pigments, such as total chlorophyll, chlorophyll-*a*, chlorophyll-*b*, and carotenoids content; and (5) plant biomass and plant growth, such as shoot dry weight, root dry weight, and length of shoot.

Alternatively, the results indicated that the addition of SNP reduced heavy metal accumulation in plant organs (shoot, stem, and root) as well as heavy metal translocation from root to shoot. However, when combined with heavy metal absorption and accumulation on the root surface, SNP enhances the phytoremediation potential of *Pleioblastus pygmaea* L. as well as soil heavy metal remediation, demonstrating that it could be used as an effective application in the phytoremediation induced process.

## Figures and Tables

**Table 1:** The content of various levels of SNP and Mn and Cr accumulation in bamboo stems, leaves, and roots. The data indicated the mean ± standard error of four repetitions. The treatments include five different concentrations of SNP, either individually or in combination with 150 μM manganese and 150 μM chromium. Different lower-case letters indicate significant differences between different concentrations of SNP used individually or in combination with 150 μM Mn and 150 μM Cr, as determined by Tukey′s test (*p* < 0.05).

SNP Concentration	Mn, and Cr Contents	Mn and Cr Accumulation (Leaves)	SNP Accumulation (Leaves)	Mn and Cr Accumulation (Stem)	SNP Accumulation (Stem)	Mn and Cr Accumulation (Root)	SNP Accumulation (Root)
µmol L^−1^	µmol L^−1^	µg g^−1^	µg g^−1^	µg g^−1^	µg g^−1^	µg g^−1^	µg g^−1^
0	0	0 ^a^	0 ^a^	0 ^a^	0 ^a^	0 ^a^	0 ^a^
0	150 µM Mn	27.42 ± 1.04 ^i^	0 ^a^	39.50 ± 0.72 ^l^	0 ^a^	45.00 ± 0.74 ^k^	0 ^a^
0	150 µM Cr	31.35 ± 0.97 ^j^	0 ^a^	43.52 ± 0.79 ^m^	0 ^a^	49.20 ± 0.84 ^l^	0 ^a^
50 µM	0	0 ^a^	25.07 ± 0.74 ^j^	0 ^a^	31.2 ± 0.77 ^h^	0 ^a^	41.30 ± 0.73 ^jk^
50 µM	150 µM Mn	19.25 ± 0.87 ^f^	11.10 ± 0.69 ^d^	28.77 ± 0.67 ^h^	16.2 ± 0.83 ^g^	31.10 ± 0.91 ^g^	23.7 ± 0.87 ^de^
50 µM	150 µM Cr	25.35 ± 1.07 ^hi^	6.2 ± 0.71 ^b^	36.35 ±0.98 ^k^	11.5 ± 0.83 ^b^	41.15 ± 0.63 ^j^	14.2 ± 0.76 ^b^
80 µM	0	0 ^a^	27.20 ± 0.42 ^k^	0 ^a^	35.1 ± 0.75 ^i^	0 ^a^	44.20 ± 0.97 ^kl^
80 µM	150 µM Mn	17.40 ± 1.10 ^def^	15.10 ± 0.80 ^f^	24.50± 0.62 ^f^	19.3 ± 0.82 ^ef^	26.15 ± 0.61 ^e^	29.6 ± 0.86 ^fg^
80 µM	150 µM Cr	23.37 ± 1.06 ^gh^	7.1 ± 0.69 ^b^	33.67 ± 0.70 ^j^	13.2 ± 0.80 ^dc^	37.12 ± 0.67 ^i^	15.9 ± 0.77 ^bc^
150 µM	0	0 ^a^	31.00 ± 0.82 ^l^	0 ^a^	37.2 ± 0.98 ^j^	0 ^a^	7.70 ± 0.79 ^lm^
150 µM	150 µM Mn	15.32 ± 1.06 ^cd^	18.12 ± 0.70 ^g^	20.20 ± 0.73 ^d^	24.6 ± 0.90 ^cb^	23.10 ± 0.50 ^d^	33.6 ± 0.75 ^ghi^
150 µM	150 µM Cr	21.65 ± 0.85 ^g^	9.1 ± 0.69 ^c^	31.45 ± 0.96 ^i^	15.5 ± 0.90 ^c^	33.15 ± 0.80 ^h^	19.8 ± 1.06 ^cd^
250 µM	0	0 ^a^	33.15 ± 0.81 ^m^	0 ^a^	42.1 ± 0.71 ^k^	0 ^a^	51.25 ± 8.12 ^mn^
250 µM	150 µM Mn	14.40 ± 1.11 ^c^	20.10 ± 0.74 ^h^	17.27 ± 0.70 ^c^	36.2 ± 0.94 ^ij^	21.25 ± 0.99 ^c^	36.5 ± 0.82 ^hij^
250 µM	150 µM Cr	18.27 ± 1.10 ^ef^	13.0 ± 0.78 ^e^	26.55 ± 0.65 ^g^	18.2 ± 0.94 ^de^	28.12 ± 0.72 ^f^	26.6 ± 0.82 ^ef^
400 µM	0	0 ^a^	36.05 ± 0.87 ^n^	0 ^a^	45.3 ± 1.03 ^l^	0 ^a^	55.30 ± 1.03 ^n^
400 µM	150 µM Mn	12.15 ± 0.62 ^b^	23.10 ± 0.79 ^i^	15.40 ± 0.72 ^b^	29.2 ± 0.96 ^h^	18.02 ± 0.75 ^b^	38.2 ± 0.88 ^ij^
400 µM	150 µM Cr	16.30 ± 1.08 ^cde^	16.1 ± 0.61 ^f^	22.30 ± 0.71 ^e^	21.2 ± 0.80 ^f^	24.22 ± 0.56 ^d^	31.2 ± 0.96 ^fgh^

**Table 2:** The effect of various SNP concentrations on chlorophyll pigments (Chl-*a*, Chl-*b*, Total Chl, and Carotenoid). The data indicated the mean ± standard error of four repetitions. The treatments include five different concentrations of SNP, either individually or in combination with 150 μM manganese and 150 μM chromium. Different lower-case letters indicate significant differences between different concentrations of SNP used individually or in combination form with 150 μM Mn and 150 μM Cr, as determined by Tukey′s test (*p* < 0.05).

Treatment	Chl-*a* (mg g^−1^ F.w.)	Chl-*b* (mg g^−1^ F.w.)	Chl *a* + *b* (mg g^−1^ F.w.)	Caratenoids (mg g^−1^ F.w.)
Control	13.20 ± 0.15 ^ghi^	11.87 ± 0.66 ^gh^	22.32 ±4.87 ^efgh^	3.36 ± 0.52 ^defg^
150 µM Mn	8.59 ± 0.42 ^ab^	6.96 ± 0.75 ^ab^	15.55 ± 1.17 ^ab^	2.02 ± 0.17 ^ab^
150 µM Cr	7.77 ± 0.23 ^a^	6.09 ± 0.56 ^a^	13.86 ± 0.47 ^a^	1.46 ± 0.54 ^a^
50 µM SNP	13.62 ± 0.28 ^hij^	13.43 ± 0.69 ^hij^	27.06 ± 0.97 ^ij^	3.57 ± 0.14 ^efgh^
50 µM SNP +150 µM Mn	10.67 ± 0.40 ^de^	9.16 ± 0.74 ^cde^	19.83 ± 0.98 ^cdef^	2.63 ± 0.16 ^bcd^
50 µM SNP +150 µM Cr	8.74 ± 0.38 ^ab^	7.96 ± 0.76 ^abc^	16.71 ± 1.12 ^abc^	2.41 ± 0.17 ^bc^
80 µM SNP	13.91 ± 0.38 ^ij^	13.90 ± 0.52 ^ijk^	27.82 ± 0.30 ^ijk^	3.67 ± 0.10 ^fgh^
80 µM SNP +150 µM Mn	11.64 ± 0.23 ^ef^	10.29 ± 0.84 ^defg^	21.93 ± 0.70 ^efgh^	2.86 ± 0.21 ^cde^
80 µM SNP +150 µM Cr	9.50 ± 0.36 ^bc^	8.21 ± 0.73 ^bc^	17.71 ± 0.94 ^bcd^	2.45 ± 0.14 ^bc^
150 µM SNP	14.17 ± 0.53 ^ijk^	15.26 ± 1.05 ^jkl^	29.43 ± 1.01 ^jk^	4.14 ± 0.55 ^h^
150 µM SNP +150 µM Mn	12.73 ± 0.24 ^gh^	11.37 ± 0.95 ^fg^	24.11 ± 1.19 ^ghi^	3.01 ± 0.16 ^cdef^
150 µM SNP +150 µM Cr	10.05 ± 0.54 ^cd^	8.62 ± 0.44 ^bcd^	18.67 ± 0.12 ^bcde^	2.48 ± 0.06 ^bc^
250 µM SNP	14.46 ± 0.19 ^jk^	15.52 ± 0.68 ^kl^	29.91 ± 0.89 ^jk^	4.01 ± 0.13 ^gh^
250 µM SNP +150 µM Mn	13.35 ± 0.32 ^hi^	12.05 ± 0.62 ^ghi^	25.40 ± 0.94 ^hi^	3.12 ± 0.09 ^cdef^
250 µM SNP +150 µM Cr	11.28 ± 0.49 ^e^	9.67 ± 0.85 ^cdef^	20.95 ± 0.37 ^defg^	2.79 ± 0.51 ^cd^
400 µM SNP	14.94 ± 0.70 ^k^	16.11 ± 0.66 ^l^	31.06 ± 1.31 ^k^	4.16 ± 0.15 ^h^
400 µM SNP + 150 µM Mn	13.56 ± 0.28 ^hij^	13.31 ± 0.88 ^hi^	26.88 ± 1.15 ^ij^	3.56 ± 0.19 ^efgh^
400 µM SNP + 150 µM Cr	12.33 ± 0.40 ^fg^	10.61 ± 0.72 ^efg^	22.95 ±1.11 ^fgh^	2.85 ± 0.16 ^cde^

**Table 3:** The effects of different concentrations of SNP on *Pleioblastus pygmaea* L. biomass in terms of shoot and root dry weights and shoot length, both individually and in combination with manganese and chromium, in comparison to the control treatment. ↑ indicates increases and ↓ indicates decreases.

SNP Levels (µM)	(Mn) and(Cr) Levels	Dryshoot Weight	Dry Root Weight	Shoot Lenght
0	150 µM Mn	33% ↓	32%↓	18% ↓
0	150 µM Cr	42% ↓	36%↓	22% ↓
50	0	10% ↑	12%↑	5% ↑
50	150 µM Mn	22% ↓	18%↓	9% ↓
50	150 µM Cr	31% ↓	29%↓	16% ↓
100	0	14% ↑	16%↑	6% ↑
100	150 µM Mn	15% ↓	12%↓	5% ↓
100	150 µM Cr	29% ↓	25%↓	14% ↓
150	0	19% ↑	8% ↑	21% ↑
150	150 µM Mn	4% ↓	1% ↓	4% ↓
150	150 µM Cr	26% ↓	11% ↓	22% ↓
250	0	24% ↑	16% ↑	25% ↑
250	150 µM Mn	1% ↑	4%↑	2% ↑
250	150 µM Cr	18% ↓	15% ↓	7% ↓
400	0	31% ↑	5% ↑	35% ↑
400	150 µM Mn	7% ↑	3% ↑	8% ↑
400	150 µM Cr	11% ↓	4% ↓	8% ↓

**Table 4:** Changes in the translocation factor and tolerance index of shoots and roots, as well as the bioaccumulation factor, in response to SNPs individually or in combination with 150 μM Mn or 150 μM Cr, when compared to the control treatment. The data indicated the mean ± standard error of four repetitions. Different lower-case letters The lowercase letters (a, b, c, d, etc.) indicate significant differences between different concentrations of SNP used individually or in combination form with 150 µM Mn and 150 µM Cr, as determined by Tukey’s test (*p* < 0.05).

Treatments	Translocation Factor (TF) (Leaves)	Tolerance Index (TI) (Shoot)	Tolerance Index (TI) (Root)	Bioaccumulation Factor (Leaves) (BAF)
Control	0.00 ± 0.00 ^a^	1.00 ± 0.00 ^ghi^	1.00 ± 0.00 ^hij^	0.00 ± 0.00 ^a^
150 µM Mn	0.69 ± 0.01 ^bc^	0.66 ± 0.04 ^ab^	0.67 ± 0.03 ^ab^	0.18 ± 0.001 ^i^
150 µM Cr	0.69 ± 0.04 ^c^	0.57 ± 0.04 ^a^	0.63 ± 0.08 ^a^	0.21 ± 0.006 ^j^
50 µM SNP	0.00 ± 0.00 ^a^	1.10 ± 0.05 ^ijk^	1.12 ± 0.10 ^klm^	0.00 ± 0.00 ^a^
50 µM SNP +150 µM Mn	0.65 ± 0.02 ^bc^	0.77 ± 0.05 ^bcde^	0.81 ± 0.02 ^cdef^	0.12 ± 0.005 ^f^
50 µM SNP +150 µM Cr	0.67 ± 0.02 ^bc^	0.68 ± 0.02 ^ab^	0.70 ± 0.01 ^abc^	0.16 ± 0.007 ^h^
80 µM SNP	0.00 ± 0.00 ^a^	1.14 ± 0.05 ^jkl^	1.16 ± 0.05 ^lmn^	0.00 ± 0.00 ^a^
80 µM SNP +150 µM Mn	0.66 ± 0.03 ^bc^	0.84 ± 0.05 ^def^	0.87 ± 0.03 ^efg^	0.11 ± 0.004 ^de^
80 µM SNP +150 µM Cr	0.67 ± 0.01 ^bc^	0.70 ± 0.05 ^bc^	0.74 ± 0.00 ^abcd^	0.15 ± 0.006 ^gh^
150 µM SNP	0.00 ± 0.00 ^a^	1.19 ± 0.02 ^kl^	1.22 ± 0.09 ^mn^	0.00 ± 0.00 ^a^
150 µM SNP +150 µM Mn	0.63 ± 0.03 ^bc^	0.95 ± 0.06 ^fgh^	0.95 ± 0.01 ^ghi^	0.10 ± 0.006 ^cd^
150 µM SNP+150 µM Cr	0.67 ± 0.01 ^bc^	0.73 ± 0.04 ^bcd^	0.77 ± 0.00 ^bcde^	0.14 ± 0.005 ^g^
250 µM SNP	0.00 ± 0.00 ^a^	1.24 ± 0.03 ^lm^	1.25 ± 0.03 ^no^	0.00 ± 0.00 ^a^
250 µM SNP +150 µM Mn	0.62 ± 0.01 ^bc^	1.01 ± 0.05 ^hi^	1.04 ± 0.02 ^ijk^	0.09 ± 0.007 ^c^
250 µM SNP +150 µM Cr	0.63 ± 0.01 ^bc^	0.81 ± 0.04 ^cde^	0.84 ± 0.02 ^defg^	0.12 ± 0.007 ^ef^
400 µM SNP	0.00 ± 0.00 ^a^	1.31 ± 0.03 ^m^	1.35 ± 0.02 ^o^	0.00 ± 0.00 ^a^
400 µM SNP +150 µM Mn	0.60 ± 0.02 ^b^	1.07 ± 0.04 ^ij^	1.08 ± 0.02 ^jkl^	0.08 ± 0.004 ^b^
400 µM SNP +150 µM Cr	0.63 ± 0.01 ^bc^	0.89 ± 0.04 ^efg^	0.91 ± 0.04 ^fgh^	0.10 ± 0.007 ^cde^

**Table 5:** The experimental design.

Experiment Treatments	Concentrations
Control	0
Mn	150 µM Mn
Cr	150 µM Cr
SNP	50 µM SNP
SNP +Mn	50 µM SNP + 150 µM Mn
SNP +Cr	50 µM SNP + 150 µM Cr
SNP	80 µM SNP
SNP +Mn	80 µM SNP + 150 µM Mn
SNP +Cr	80 µM SNP + 150 µM Cr
SNP	150 µM SNP
SNP +Mn	150 µM SNP + 150 µM Mn
SNP +Cr	150 µM SNP + 150 µM Cr
SNP	250 µM SNP
SNP +Mn	250 µM SNP + 150 µM Mn
SNP +Cr	250 µM SNP + 150 µM Cr
SNP	400 µM SNP
SNP +Mn	400 µM SNP + 150 µM Mn
SNP +Cr	400 µM SNP + 150 µM Cr

## Data Availability

The data presented in this study are available in article.
